# Study Protocol: establishing good relationships between patients and health care providers while providing cardiac care. Exploring how patient-clinician engagement contributes to health disparities between indigenous and non-indigenous Australians in South Australia

**DOI:** 10.1186/1472-6963-12-397

**Published:** 2012-11-14

**Authors:** Yvette L Roe, Christopher J Zeitz, Bronwyn Fredericks

**Affiliations:** 1 School of Health Sciences, University of South Australia, Adelaide, Australia; 2 Clinical Head of the Division of Medicine, The Queen Elizabeth Hospital, Woodville, Australia; 3 Central Queensland University, Rockhampton, Australia

**Keywords:** Patient-clinician engagement, Qualitative, Cardiovascular disease, Focus groups, Indigenous methodology, Oceanic, Cardiac care

## Abstract

**Background:**

Studies that compare Indigenous Australian and non-Indigenous patients who experience a cardiac event or chest pain are inconclusive about the reasons for the differences in-hospital and survival rates. The advances in diagnostic accuracy, medication and specialised workforce has contributed to a lower case fatality and lengthen survival rates however this is not evident in the Indigenous Australian population. A possible driver contributing to this disparity may be the impact of patient-clinician interface during key interactions during the health care process.

**Methods/Design:**

This study will apply an Indigenous framework to describe the interaction between Indigenous patients and clinicians during the continuum of cardiac health care, i.e. from acute admission, secondary and rehabilitative care. Adopting an Indigenous framework is more aligned with Indigenous realities, knowledge, intellects, histories and experiences. A triple layered designed focus group will be employed to discuss patient-clinician engagement. Focus groups will be arranged by geographic clusters i.e. metropolitan and a regional centre. Patient informants will be identified by Indigenous status (i.e. Indigenous and non-Indigenous) and the focus groups will be convened separately. The health care provider focus groups will be convened on an organisational basis i.e. state health providers and Aboriginal Community Controlled Health Services. Yarning will be used as a research method to facilitate discussion. Yarning is in congruence with the oral traditions that are still a reality in day-to-day Indigenous lives.

**Discussion:**

This study is nestled in a larger research program that explores the drivers to the disparity of care and health outcomes for Indigenous and non-Indigenous Australians who experience an acute cardiac admission. A focus on health status, risk factors and clinical interventions may camouflage critical issues within a patient-clinician exchange. This approach may provide a way forward to reduce the appalling health disadvantage experienced within the Indigenous Australian communities.

## Background

Acute Coronary Syndrome (ACS) represents one of the most common causes of acute medical admissions to Australian hospitals [1,2]. Patients with ACS have varying medical histories, diverse clinical presentations and are likely to experience a second serious cardiac event; they require acute in-hospital clinical care and experience high mortality rates [3]. Management of ACS is targeted towards identifying those patients at higher risk of subsequent events and reducing or avoiding such events through revascularisation of the culprit plaque, where appropriate, and medical therapy to stabilise other at risk plaques [4].

The incidence of ACS has increased and the outcomes of care for ACS have shown smaller improvement for Aboriginal and Torres Strait Islander^a^ Australians compared to their non-Indigenous counterparts [5-12]. A national retrospective study on ACS management in Australia reported three striking failures and policy issues that were systemic: an evidence practice-gap; and concern about the capacity of the treating hospital and the continuity of care available [13]. Many contributing factors lead to less than optimal health outcomes for Indigenous patients; these factors often lie outside the health system itself [1,5,6,14-16]. For example, studies that compare Indigenous and non-Indigenous patients with ACS and or chest pain are inconclusive about the reasons for the differences in-hospital and survival rates. It has been argued that the difference in treatment and outcomes may be the result of more subtle systemic practices, not necessarily ill-intentioned but still discriminatory, and almost invisible within an individual patient-clinician encounter [14]. These findings highlight the importance of quantifying and qualifying patient-clinician engagement as a possible explanation for the differential between Indigenous and non-Indigenous patients admitted to hospital for an acute cardiac event. A focus on health status, risk factors and clinical interventions may camouflage critical issues within a patient-clinician exchange. Broadening our understanding of the Indigenous in-hospital disparities and mortality, to include patient-clinician engagement may provide opportunities to close the gap in health attainment.

This Study Protocol identifies the gaps in knowledge pertaining to patient-clinician engagement, states the research aims and questions, describes the types of people that may participate in the research, outlines the methodology and methods used as well as highlighting the limitations of the study. The Study Protocol also situates this study within a boarder research program that explores the potential drivers that may influence the differential in health care and health status experienced by Indigenous Australians compared to non-Indigenous Australians who experience an acute cardiac event.

### Conceptual model

**Figure 1:**
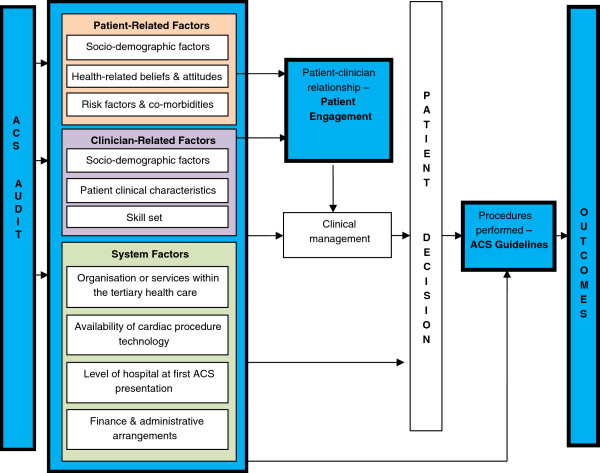
Conceptual diagram of the research.

Patient-clinician engagement is shaped by a range of factors, not only to do with the agents’ personality and socioeconomic circumstances, but also the diagnosis and proposed management, and the context of the medical encounter. Influencing factors may present as a single factor or may have aggregated and distributive attributes. Figure 1 illustrates the overall conceptual diagram for the research.

### Literature

National and international studies suggest three key areas influence the patient-clinician relationship: reflective clinical practice, a shared understanding and integration of health care; and cultural safety which lays the foundation for this interface.

### Cultural safety

Creating a culturally safe^b^ environment involves health professionals undergoing a reflective process of understanding the self and others. For example, one’s review of ontology (assumptions about the nature of reality), epistemology (the ways of knowing that reality), and axiology (the nature of values) [17-19]. When health professionals become aware of how their understanding of the ‘other’ influences their clinical practice, they may facilitate opportunities for patients to engage *‘safely’* in the patient-clinician relationship [20].

In Australia, studies that described the interaction between Indigenous patients and clinicians illuminate many of the nuances of the patient-clinician interface. Miscommunication, feelings of disempowerment, mistrust and racism were reported barriers to patient engagement [15,20,21]. A summary of the factors highlighted in the literature that influence patient-clinician engagement and adverse consequences is outlined in Table 1. This list strongly suggests that patient-clinician engagement may be compromised in the case of Indigenous patients.

**Table 1:** Factors that influence patient-clinician engagement and the adverse consequences

**Concept**	**Features**	**Adverse consequences**
Cultural Safety	· Health professional are self-aware of their ontology, epistemology and axiology.	Actions that diminish, demean or disempower the cultural identity and well-being of an individual is unsafe clinical practice [3,4].
	· An environment that facilitates and nurtures relationships	Patients viewed the medical system as cold, indifferent and inflexible [2]
	· Health services do not comprise the patients legitimate cultural rights, views and values.” [1]	
	· High staff turnover [2]	
(Mis) Communication	· Language (verbal and non-verbal),	· May result in misdiagnosis; ineffective and inefficient clinical management; and marginalisation of the patient [6–8].
	· Rules, conventions and etiquette;	
	Communication between the community primary health care provider and the tertiary institution [5]	· An inefficient model of care i.e. ‘no shows’ in patient travel and patient has limited understanding of their clinical care [5].
(Dis) Empowerment	· A distressing patient journey [5]	· Patients feel disempowered, discriminated by their race and clinicians show a lack of empathy toward them [2,3,5].
	· Financial burden	
	· Language barriers	
	· Lack of culturally appropriate resources	
	· Inadequate pre-operative preparation and post-operative follow-up.	
	· Lack of cognitive control [2,3].	
(Mis) Trust	· Informed by a whole of life experience; which included systemic oppression and discrimination with societal institutions (particularly justice and education settings).	· In response to racist treatment people felt ashamed, humiliated, powerless and fearful; which in turn contributed to the lack of trust [3].
	· In an individual encounter.	
Biomedical Model	· The dominance of medical language used to explain clinical diagnosis, management and long-term care.	· Removes the opportunity to construct a shared understanding of health care [20].
	· Marginalisation of the patience preferred language or knowledge [2,20].	· Patients feel alienated and less likely to participate with the recommended care [16]

There is great scope for improvement in building a shared understanding in the patient-clinician interface. Australian studies reported a number of factors that influenced the quality of the patient-clinician relationship. The most common factor identified is miscommunication between the patient-clinician. Miscommunication often goes unrecognised, especially with regard to diagnosis, treatment and prevention [21]. Possible solutions that may assist to overcome these barriers include: increasing the Indigenous health workforce; increasing access to interpreter services; and engaging with patients throughout the patient pathway (pre-operative; post-operative; long-term and secondary prevention). These possible solutions may contribute to ensuring culturally appropriate health education and mandatory training in cultural safety [15,16,21]. The experiences described are not unique to Indigenous people; nonetheless they provide insights into the complexities that Indigenous people have to overcome when seeking health care.

### Patient-clinician engagement: synergetic partnership

It is hypothesised that patient-clinician engagement is instrumental for improving health care and results in improved health outcomes. For the purpose of this study, patient-clinician engagement is explored and defined at the micro level. Patient-clinician engagement is the two-way interface between patient and clinician (individually and systematically). Patient-clinician engagement can manifest as an outcome but fundamentally it is the process that defines the outcome*.*

The patient-clinician interface is framed by interrelated variables such as: gender; age; personal heterogeneities (both patient and clinician); family and professional responsibilities; cultural orientation; socioeconomic position; lived experience; and respective cultural paradigms. The interface is further contextualised by, environmental diversities, social climate and differences in relational perspectives. Engagement is not homogeneous and is neither constant nor in any sense automatic. Patient-clinician engagement is about relationships. Essentially, patient-clinician engagement is the synergy between clinical competence, cultural respect and the shared understanding that occurs when health care providers’ acknowledge and nurture the wellbeing of the patient. Failure to invest in this relationship may result in adverse outcomes such as potentially life-threatening consequences for the patient and policy implications for health care providers [3,15].

#### Methodology

##### Indigenous framework

Adopting an Indigenous framework identifies the limitation of Western paradigms to adequately portray Indigenous realities, knowledge, intellects, histories and experiences. This study will apply an Indigenous framework to describe the interaction between Indigenous patients and clinician clinical encounters during an acute cardiac admission.

Indigenous knowledges and health status are often problematised and pathologised [18,22]. Indigenous people are frequently positioned as being dysfunctional and challenging. This perpetuates a body of health research where Indigenous knowledges are disregarded. This deficit approach obscures the survival and resistance strategies employed by Indigenous Australians for over 220 years. Indigenous methodology makes it explicit that the study will be viewed through a culturally-specific lens that privileges Indigenous realities by taking account of Indigenous epistemologies and ontologies [18].

By using an Indigenous methodology we can situate Indigenous peoples as the subjects of their experiences rather than objects of the research [23]. Indigenous Australians think and interpret the world and its realities in different ways to non-Indigenous Australians because of their experiences, histories, cultures and values, which in turn influences their patterns of morbidity, mortality and health outcomes. Indigenous ways of knowing is a continuum because context is important and this is particularly important in an area where efforts from non-Indigenous researchers towards ‘knowing’ have often been intrusive and exploitative [22,23]. Finally, cultural safety, cultural respect, cultural relevance and world view alignment is the nucleus to an Indigenous framework [24].

#### Research scope

##### Research questions

1. How do Indigenous and non-Indigenous patients who were admitted for an acute cardiac event describe patient-clinician engagement?

2. How do clinicians who provide cardiac care to patients describe Indigenous and non-Indigenous patient-clinician engagement?; and

3. How can patient-clinician engagement improve cardiac care hospital admission and transition into secondary health care?

##### Research aims

1. To describe patient-related factors, clinician-related factors and system factors that influence the patient-clinician engagement for Indigenous and non-Indigenous patients diagnosed and treated for an acute cardiac event in South Australia; and

2. To identify areas of potential improvement that may support patient-clinician engagement during and after admission for an acute cardiac event i.e. into secondary and rehabilitative cardiac care.

## Methods

A triple layered designed focus group will be the format to discuss patient-clinician engagement. Seeking opinions using focus groups provides an effective platform for developing a holistic and contextualised understanding of the diverse factors in a complex health setting [22,25].

Focus groups will be arranged by geographic clusters i.e. metropolitan and a regional centre. Patient informants will be identified by Indigenous status (i.e. Indigenous and non-Indigenous) and the focus groups will be convened separately. The health care provider focus groups will be convened on an organisational basis i.e. state health providers and Aboriginal Community Controlled Health Service Sector (Table 2). There will be nine focus groups comprising five to seven participants. Participants may have similar social and cultural backgrounds or have similar experiences pertaining to their cardiac diagnosis, management and clinical experience.

**Table 2:** Focus Group Questions

**Question Type**	**Purpose**	**Example**	
		**Patient Informant**	**Clinician Informant**
Opening	Participants get acquainted & feel comfortable	Please tell us your name and how are you feeling today?	Please tell us your name and where you work?
Introductory	Begins discussion of topic	How has having a heart problem influenced your life?	How long have you been employed in cardiac care?
Transition	Moves smoothly and seamlessly into key questions	I would like you to reflect when you became unwell because of your heart, when you were admitted to hospital and after you left hospital.	We often use the term patient journey, however there are many components to it i.e. clinical presentation, guidelines, risk factors, patient involvement etc.
		Are there any specific things about the how you got along with the health care providers or the care you received that stands out in your mind? How would you describe your relationship with the people who provided your care?	Are there any specific things about that you have done during your interaction with patient that you felt made it a good interaction that is critical to providing cardiac care? E.g. imparting facts in a way that there is a shared meaning?
Key	Obtains insight into areas of central concern in the study	Do you feel that you were able to establish trust and rapport with the people who provided your care?	When I say the term ‘patient-clinician engagement’ what things come to mind?
		Do you feel that you were able to talk easy to the health staff, especially if you had concerns about your heart or things that worried you?	Is it a term that you’ve heard of and that you are familiar with?
			Do you felt you developed trust and rapport with the patients?
		What things made you feel like you had a good relationship with the people who provided your health care?	How do you build trust, rapport and a shared understanding with patients?
		When you left hospital, how easy was it to gets health care so you could look after your heart? i.e. seeing the doctor or specialists, rehabilitation, getting medication etc.	Where do you think trust, rapport and understanding falls downs when you are caring for patients?
			Do you have a specific strategy when caring for Indigenous patients?
		What are the things that may influence or stop you looking after your heart?	Can you provide some examples of how care maybe different for Indigenous and non-Indigenous patients?
		What can health care providers do to help you look after your heart?	What can health professionals do to encourage patient-clinician engagement? Is this different for Indigenous and non-Indigenous patients?
Ending	Helps the researcher determine where to place emphasis and bring closure to the discussion.	Moderator to summarise key discussion points.	
		Is this an adequate summary of what was said here?	
		We are trying to make the patient journey better.	
		What advice would you have for us?	

1. Houston S. Aboriginal cultural security. Perth: Health Department of Western Australia2001.

2. Shahid S, Finn L, Thompson S. Barriers to participation of Aboriginal people in cancer care: communication in the hospital setting. Medical Journal of Australia. 2009;190:574–9.

3. Gallaher G, Ziersch A, Baum F, Bently M, Palmer C, Edmondson W, et al. In our own backyard: Urban health inequities and Aboriginal experiences of neighbourhood life, social capital and racism. Adelaide: Flinders University2009.

4. Ramsden I. The Treaty of Waitangi and cultural safety: The role of the treaty in nursing and midwifery education in Aotearoa. Wellington: Nursing Council of New Zealand1996.

5. Lawrence M, Dodd Z, Mohor S, Dunn S, de Crespigny C, Power C, et al. Improving the Patient Journey: Achieving Positive Outcomes for Remote Aboriginal Cardiac Patients. Darwin: Cooperative Research Centre for Aboriginal Health2009.

6. Lowell A, Brown I, Marrnanyin B, Flack M, Christie M, Snelling P, et al. Sharing the true stories, Improving communication between health staff and Aboriginal patients, STAGE 1 Report. Darwin: Cooperative Research Centre for Aborignal Health2005.

7. Brown A, D., Morrissey M, J., Sherwood J, M. Uncovering the Determinants of Cardiovascular Disease among Indigenous People. Ethnicity & Health. 2006;11 [2]:191–210.

8. Cass A, Lowell A, Christie M, Snelling P, L., Flack M, Marrnganyin B, et al. Sharing the true stories: improving communication between Aboriginal patients and health care workers. Medical Journal of Australia. 2002 20 May 2002;176:466–70.

### Data collection: yarning

Yarning will be used as a research method to facilitate discussion. Yarning as a strategy assists in decolonising, re-positioning and supporting Indigenous research methods as well as embedding itself within the Indigenous framework [18,23-25]. Yarning is in congruence with the oral traditions that are still a reality in day-to-day Indigenous lives. Yarning also acknowledges the relatedness of past and present and also the future [26].

While widely recognised in an informal social setting, Yarning in the research context is somewhat different. That is, while the technique is relaxed and interactive it is also purposeful with a defined beginning and end. Bessarab and Ng’angu suggests that yarning as a method enables the researcher and participant(s) to develop an informal relationship whereby information can be shared and exchanged between two or more people either socially or more formally [26,27].

Yarning as a method has proven effective when applied within a number of settings involving Indigenous people and within policy development [25-28]. Sharing stories using Yarning is a means of knowing and sharing knowing [28]. In its essence, yarning is a two-way process where people share knowledge. The focus group data collection method is predicated on engaging all participants in a safe, relaxed and inviting manner so they are able to share their lived experience. This will involve selecting a venue that is accessible, inviting and relaxing, ensuring that the seating arrangements encouraging open conversations, provision of food, informal introductions of participants, while also accommodating gender, age of patients and diverse cardiac workforce.

A series of questions will be asked to the group to gain a better understanding of patient-clinician engagement both from a patient and clinician perspective. A broad question will be asked of patient participants to begin the interview: *“How has having a heart problem changed your life?”* The clinician focus group will also commence with a broad question, “*How long have you worked in cardiac care?”* The moderator may pose additional questions and offer observations for comment to keep the discussion focused, or to clarify information provided by participants. The questions are open-ended and semi-structured (Table 3).

The focus groups provide the formal structure for the participant gathering. This might include venue, catering, recording of narratives, and group composition. However, the richness of the data will be drawn out by the facilitators’ ability to negotiate a contextually based conversation with participants that fosters an interactive environment. The application of Yarning as a method allows the facilitator to ask purposefully targeted questions about the participants’ cardiac care experience while also establishing a safe, informal and respectful relationship with these participants. The interaction between the facilitator and participants will be informed by the semi-structured questions in a conversational space where there is two-way interaction between the facilitator and participants.

There is also social protocols and conventions involved in conversing that need to be acknowledged and embraced in this dialogical process [26]. In addition, listening (not just hearing) and valuing the context the participants’ experience is as equally as important as the questions posed. Finally, there is a multiplicity of factors that influence patient-clinician engagement and capturing the subtle nuances in how participants may share their lived experience is pivotal. The facilitator is consciously applying the every-day-conversational method while also seeking an insight into the patient-clinician interaction by not only stating the outcome but also illuminating the processes that define the outcomes. Other authors who have applied this method have acknowledged that participants may stray from the posed question resulting ‘messy text’ [26,29]. To limit the potential of this occurring the facilitator will be trained to acutely listen to the yarn, respect/embrace conversational protocols and gently refocus the discussion back to the research question. Underlying the interaction between the facilitator and participant is a specific research question that includes a dynamic process, of data collection and data analysis.

#### Participants

A Participant Information Sheet or a customised flip chart (to assist with literacy) in plain English will be provided to potential participants. The Information Sheet outlines what is required of participants as well as any possible risks to them resulting from their participation. The Participant Consent Form will be in plain English and participants are free to withdraw at any time, without affecting their status now or in the future. Consent will be obtained from all participants.

### Inclusion criteria

Patient informants aged 18–75 years discharged from hospital between 1 January 2008 and 31 December 2011; diagnosed with an acute cardiac event and South Australian residents. Indigenous status will be determined on the basis of self-identification; and

Clinical informants aged 18 years (and over) employed to provide cardiac care to patients within the continuum of cardiac care i.e. transition of patients from acute care to long term care and secondary prevention.

### Recruitment

Purposive sampling will be used to recruit informants to the study. This approach requires the deliberate selection of specific individuals (patients and clinicians) because of the crucial information they can provide on engagement, both as a process and an outcome during an acute cardiac event [29,30]. This approach has proven to be effective with Indigenous people [26,31]. Recruited informants are thereby best placed to provide information-rich narratives into patient-clinician engagement.

Three public cardiac care health providers and two Aboriginal Community Controlled Health Services in South Australia have been approached and agreed to assist with the recruitment of Indigenous and non-Indigenous participants. Discussion with key stakeholders and displaying of information flyers will occur at each site to attract participants.

Participation in the study is recognised by compensating participants for their time, especially if they incurred financial or emotional expense to participate such as: child care; travel; and the possible apprehension of talking about their personal experiences [29]. Patient participants will be provided with a $30 gift voucher to use at a local shopping retailer on completion of the focus group.

#### Analysis

The study will use the group, rather than the individual group members, as the unit of analysis. Group interaction through the process of yarning will take into account and the levels of consensus generated by the different topics both between and within groups will be assessed. The analysis will systematically elicit themes in relation to the research questions.

**Figure 2:**
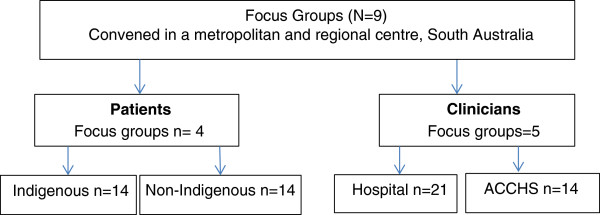
Triple layered designed focus groups: Locations and Composition.

The focus groups’ transcripts are the primary source of the data analysis. The transcript will also include boarder communication traits that occur during the yarning process such as tones, silences and body language. After immersion, the data will be coded and categorised into preliminary themes (Figure 2) [32]. Emerging themes and patterns will be identified and final conclusions developed and verified by another member of the research team. The analysis will be done as a direct reflection of the conceptual model and study question.

All analyses will be facilitated by the use of QSR International NVivo software, version 9 (QSR International). NVivo will be used to manage data, manage ideas, query data, illustrate models and report from the data [33].

### Validity

The study will draw on multiple sources of evidence such as the transcripts, field notes and a reflective journal. Participants will have the opportunity to view and validate the group transcript before the analysis. Participants may delete, add or modify any of their content.

An independent review of the interview transcripts will occur. The reviewer (PhD student supervisor) will be provided with a sample of interview questions and asked to code and categorise the data. There will be an assessment of the level of congruence between the researcher and independent reviewer in coding and categorising data. The higher the congruence between the researcher and reviewer the more confident one can be that internal validity is met.

### Ethical considerations

#### Health consumer advocacy

The Participant Information sheet outlines the risks of participating in the research i.e. the process of sharing experiences has the potential to raise many emotions such as anger or grief and yet may be equally healing. If a participant becomes distressed during the discussion they are encouraged to seek advice from their doctor or local health centre. The researcher will provide information about the Hospital Consumer Adviser or Health Consumer Advocate and provide assistance with initial contact as required.

#### Ethics approval

The University of South Australia’s Human Research Ethics Committee and the Aboriginal Health Council Research Ethics Committee approved the research. The study design incorporates the underlying principles in the NHMRC Road Map: A Strategic Framework for Improving Aboriginal and Torres Strait Islander Health. That is, the research aims to add practical value to Indigenous peoples and their service providers, through building Indigenous research workforce capacity while also embracing community involvement in the development, conduct and communication of research [34].

#### Cultural protocol for conducting research

The principal researcher is Aboriginal, a Njikena Yawuru woman from the West Kimberley, Western Australia. The role of the researcher requires her to unpack key elements of the research process. Firstly, as an ‘Outsider’ to the South Australian Aboriginal community this process requires certain terms of reference to work (i.e. working in someone else’s Country). To adhere to Aboriginal cultural protocols and to demonstrate the respect of undertaking research on another Aboriginal nation’s sovereign land, a Cultural Protocol for Research was negotiated between the principal researcher and the Aboriginal Health Council of South Australia. The Protocol outlines principles for conducting the research; cultural mentoring; and using Indigenous knowledge and researcher accountability. The Protocol is informed by Dr Karen Martins’ seminal text *‘Please Knock before you enter: Aboriginal regulation of outsiders and the implications for research’*[25]. The Protocol also includes the role of the Indigenous Reference Group who will assist in the development of the focus group and interpretation of the data. This will be achieved by conducting a collaborative workshop between the researcher and Reference Group as a means of ensuring adherence of cultural protocols and research validity.

### Limitations

The methods applied will result in analytic generalisations. The study does not represent the views of all patients who experience an acute cardiac event or the entire cardiac health workforce. This research highlights the significance of particular contexts and settings. The design, analysis and reporting of this research intends to provide a sufficiently detailed account and analysis to enable others to determine whether there are other circumstances to which these findings may be applicable or replicate this study elsewhere [35].

The limitations are:

1. Due to the nature of group discussion, some participants may conform with the responses of other members in the group even though they might not agree [36];

2. The complexity of unpacking what is understood by the term patient-clinician engagement;

3. The double burden of engaging the ‘disengaged’;

4. To identify and address rival explanations for the findings;

5. To account for bias, poor recall and poor or inaccurate articulation; and

6. To explain the multi-factorial confounders along patient-clinician pathway.

## Discussion

This study is nestled in a larger research program that explores the drivers to the disparity of care and health outcomes for Indigenous and non-Indigenous Australians who experience an acute cardiac admission. An examination of the level of guideline concordance may provide insight into the application of evidenced-based guidelines and the capacity of the treating hospital. To ensure a comprehensive understanding of possible drivers of the disparity between Indigenous and non-Indigenous patients, intimate knowledge about the interface between the patient and clinician is needed.

Applying an Indigenous framework, using focus groups and Yarning as a method, seeks to ensure that the research is conducted rigorously, is respectful and culturally safe for all participants. This research moves away from a deficit model that currently describes Indigenous health knowledge and reorients the discussion toward a resilience and asset model of health care. This approach may provide a way forward to reduce the appalling health disadvantage experienced within the Indigenous Australian communities.

### Ethics committee approval

University of South Australia Human Research Ethic Committee.

Aboriginal Health Research Ethics Committee.

## Endnotes

^a^Aboriginal and Torres Strait Islander Australians will now be referred to as Indigenous Australians.

^b^Cultural safety also includes cultural safety, cultural security and cultural respect.

## Abbreviations

ACS: Acute Coronary Syndrome; NHMRC: National Health and Medical Research Council.

## Competing interests

The authors declare that they have no completing interests.

## Authors’ contribution

YLR designed the study, negotiated support from participating health services, negotiated the Cultural Protocol for Research between herself and the Aboriginal Health Council of South Australia and drafted the manuscript. CJZ provided intellectual planning of the research, contributed to the research design and reviewed/edited the manuscript. BF participated in the designing the methodological framework of the study and thematic analysis. All authors read and approved the final manuscript.

## Pre-publication history

The pre-publication history for this paper can be accessed here:

http://www.biomedcentral.com/1472-6963/12/397/prepub
